# Tuning of Mechanical Properties in Photopolymerizable Gelatin-Based Hydrogels for *In Vitro* Cell Culture Systems

**DOI:** 10.1021/acsapm.2c01980

**Published:** 2023-01-27

**Authors:** Regina Pamplona, Sandra González-Lana, Pilar Romero, Ignacio Ochoa, Rafael Martín-Rapún, Carlos Sánchez-Somolinos

**Affiliations:** † Aragón Institute of Nanoscience and Materials (INMA), Department of Organic Chemistry, CSIC-University of Zaragoza, C/ Pedro Cerbuna 12, 50009Zaragoza, Spain; ‡ BEONCHIP S.L., CEMINEM, Campus Río Ebro. C/ Mariano Esquillor Gómez s/n, 50018Zaragoza, Spain; § Tissue Microenvironment (TME) Laboratory, Aragón Institute of Engineering Research (I3A), University of Zaragoza, C/ Mariano Esquillor s/n, 50018Zaragoza, Spain; ∥ Centro de Investigación Biomédica en Red de Bioingeniería, Biomateriales y Nanomedicina, Instituto de Salud Carlos III, 50018Zaragoza, Spain; ⊥ Institute for Health Research Aragón (IIS Aragón), Paseo de Isabel La Católica 1-3, 50009Zaragoza, Spain; # Departamento de Química Orgánica, Facultad de Ciencias, Universidad de Zaragoza, C/ Pedro Cerbuna 12, 50009Zaragoza, Spain; ∇ Aragón Institute of Nanoscience and Materials (INMA), Department of Condensed Matter Physics (Faculty of Science), CSIC-University of Zaragoza, C/ Pedro Cerbuna 12, 50009Zaragoza, Spain

**Keywords:** gelatin, hydrogel, thiol-ene, photopolymerization, nanoindentation, colorectal

## Abstract

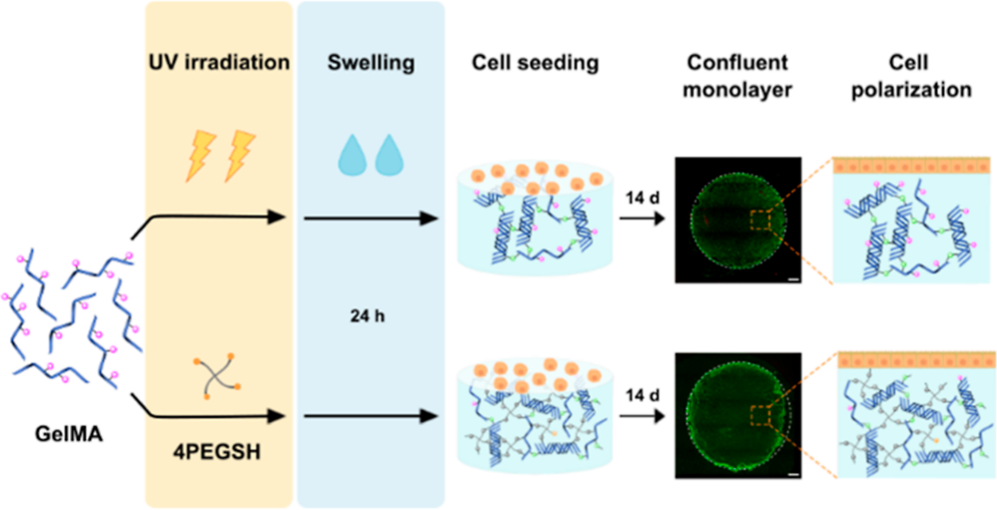

The mechanical microenvironment plays a crucial role in the evolution of colorectal cancer, a complex disease characterized by heterogeneous tumors with varying elasticity. Toward setting up distinct scenarios, herein, we describe the preparation and characterization of gelatin methacrylamide (GelMA)-based hydrogels *via* two different mechanisms: free-radical photopolymerization and photo-induced thiol-ene reaction. A precise stiffness modulation of covalently crosslinked scaffolds was achieved through the application of well-defined irradiation times while keeping the intensity constant. Besides, the incorporation of thiol chemistry strongly increased stiffness with low to moderate curing times. This wide range of finely tuned mechanical properties successfully covered from healthy tissue to colorectal cancer stages. Hydrogels prepared in phosphate-buffered saline or Dulbecco’s modified Eagle’s medium resulted in different mechanical and swelling properties, although a similar trend was observed for both conditions: thiol-ene systems exhibited higher stiffness and, at the same time, higher swelling capacity than free-radical photopolymerized networks. In terms of biological behavior, three of the substrates showed good cell proliferation rates according to the formation of a confluent monolayer of Caco-2 cells after 14 days of cell culture. Likewise, a characteristic apical-basal polarization of cells was observed for these three hydrogels. These results demonstrate the versatility of the presented platform of biomimetic materials as *in vitro* cell culture scaffolds.

## Introduction

The extracellular matrix (ECM) is a three-dimensional network mainly composed of water, proteins, proteoglycans, and polysaccharides which provides both physical scaffolding and biochemical cues regulating cell processes such as differentiation, adhesion, and migration, among others.^1^ It is well established that cells and their surrounding ECM communicate bidirectionally^1,2^ and as a result, the ECM is not a static system of biopolymers, but it is under constant remodeling performed by cells.^2,3^ However, the dysregulation of normal ECM homeostasis leads to different types of disease.^3^ Cancer progression is characterized by a stiffening process because of an aberrant deposition of biomolecules such as collagen^4^ and hyaluronan,^5^ but still many of the processes behind remain unknown. Aiming to better understand tumor growth mechanisms as well as cell–matrix interactions, scaffolds with tunable mechanical and biochemical properties are required.

Among the numerous culture models described in bibliography,^6,7^ it is known that hydrogels have great potential to mimic cell microenvironments owing to their tunable properties.^8^ Hydrogels are macromolecular networks of hydrophilic nature thus capable of incorporating high water contents, which is a key feature for nutrient and residue transport and hence cell survival.^9^ Matrix remodeling, cell adhesion, and diffusivity can also be adjusted through degradable motifs, recognition sequences, and the mesh size of these constructs, respectively, to create an artificial ECM.^10^

Since the first approaches to biomimetic networks, a plethora of customizable microenvironments has been explored so far.^11^ Collagen, hyaluronic acid, and fibronectin scaffolds are natural protein- or polysaccharide-based materials, and their ubiquitous use is due to intrinsic advantageous attributes including biocompatibility, cell adhesion capability, and non-toxicity.^11,12^ Likewise, gelatin is a widespread biomaterial which is obtained from the denaturation of collagen^13^ and contains the arginine-glycine-aspartic acid (RGD) sequence promoting cell adhesion, proliferation, and differentiation. Besides, gelatin displays a unique thermo-reversible gelation as a consequence of a conformational transition from random coil to triple helix.^13^ This aggregation is stabilized through intermolecular hydrogen bonding, and the resulting physical crosslinking is proved to be associated with superior mechanical properties.^14^ In this context, gelatin methacrylamide (GelMA) hydrogels emerged as promising materials 20 years ago when Van Den Bulcke *et al.* derivatized gelatin with methacrylamide side groups.^15^ GelMA hydrogels became attractive due to the peptidic backbone, which provides good cell adhesion sites,^16^ as well as to their ability of undergoing photopolymerization to form covalently crosslinked networks.^17^

Photopolymerization has been a broadly applied method to *in situ* prepare tridimensional networks with well-controlled properties.^18,19^ Photopolymerization allows for spatiotemporal control over hydrogel formation by using photomasks to pattern local areas^20^ and applying well-defined irradiation doses. In widely used photoinduced chain-growth network formation, typically, photogenerated radicals propagate through monomers or macromers bearing multiple double bonds to lead to kinetic chains that become covalently crosslinked. Chain-growth photopolymerization has been frequently studied because of solvent-free requirements and mild reaction conditions.^21^ This hydrogel formation approach has demonstrated to be suitable for the modulation of mechanical properties such as hydrogel stiffness, an essential parameter that influences cell behavior.^16^ A number of research works with photocrosslinked gelatin hydrogels have shown that substrate rigidity can impact chondrogenic phenotype,^16^ endothelial differentiation,^22^ cell proliferation, and migration.^23^ Besides, this tuning has been approached through several crosslinking parameters such as the initiator concentration,^21^ the methacrylation degree of GelMA,^16^ the macromer concentration,^22^ the UV exposure,^24^ and the curing time.^25^ Given that native and tumor tissues can vary in stiffness as a result of ECM composition changes happening during cancer development,^26^ there has been an increasing concern about setting up different matrix scenarios. For this reason, it is highly valuable to explore rapid techniques such as free-radical photopolymerization that enable to precisely control biophysical properties of hydrogels.

Photoinduced thiol-based click reactions, on the other hand, are considered to be a powerful alternative in the biomedical field to achieve spatiotemporal control over mechanics.^27^ Thiol-ene reaction owns many of the advantages of click chemistry, including robustness, simplicity, high selectivity, quantitative yielding, and insensitivity to oxygen or water.^27^ Owing to these attributes, there has been large interest in the last decade about the preparation of gelatin hydrogels introducing thiol reactivity toward electron-rich/electron-poor carbon–carbon double bonds.^28−30^ Some reports have been published considering the research in which gelatin contains the thiol moiety,^31,32^ although there is a wider variety in case gelatin contains the double-bond.^33−35^ Thus, thiol-based click chemistry using norbornene-functionalized gelatin has been thoroughly described^36−39^ and mostly applied in biofabrication.^40−42^ As for the thiol-methacrylamide system, few studies have been found and only macromer concentration has been deeply investigated about its influence on mechanical properties and cell response.^43^ In those relevant studies, either the reaction involves a macromer also on the thiol side—gelatin, heparine, lignosulfonate—or the thiol is added after the acrylate photopolymerization has taken place.^44−47^ Other researchers have used in the same material crosslinking reactions—thiol-yne or dynamic covalent chemistry—additional to acrylate photopolymerization and thiol-ene reaction, which make interpretation of the structure–property relationships more challenging.^44,48^

Herein, we present a platform of GelMA-based photopolymerizable hydrogels with tunable mechanical properties for culture of human colon carcinoma cell line (Caco-2). We have generated scaffolds for *in vitro* intestinal models using on one hand hydrogels prepared by free-radical photopolymerization of GelMA. On the other hand, hydrogels have also been prepared combining the photopolymerization of GelMA with the photoinduced thiol-ene reaction of GelMA with a thiol-containing poly(ethylene glycol) (PEG) crosslinker. We aimed first at controlling the stiffness of the hydrogels by changing the time of UV-light exposure instead of macromer concentration and second to shorten the curing time by using concurrent thiol chemistry in order to diminish the dose of potentially harmful UV radiation. These strategies have been tested through the evaluation of Young’s modulus, gel fraction, and swelling behavior. Most importantly, the suitability of the materials as scaffolds for intestinal epithelium models has been investigated.

## Materials and Methods

### Materials

Gelatin from porcine skin (Type A, 300 Bloom), methacrylic anhydride (MAA) (94%), dialysis tubing cellulose membranes (MWCO: 12–14 kDa), 3-(trimethylsilyl)propionic-2,2,3,3-*d*_4_ acid sodium salt (TMSP), 2,4,6-trinitrobenzenesulfonic acid 5% w/v solution (TNBS), sodium *n*-dodecyl sulfate 20% w/v solution (SDS), glycine (ReagentPlus, ≥99%), and photoinitiator 2-hydroxy-4′-(2-hydroxyethoxy)-2-methylpropiophenone, also known as Irgacure 2959 (I2959), were purchased from Sigma-Aldrich. Methanol was purchased from PanReac AppliChem ITW Reagents. Deuterium oxide was purchased from Eurisotop. 4-arm poly(ethylene glycol) thiol (4PEGSH) was purchased from JenKem, USA. Poly(dimethylsiloxane) (PDMS) elastomer was prepared from Sylgard-184 (Dow Corning). Glass coverslips (thickness: 0.16 mm) for immunostaining visualization were purchased from Marienfeld GmbH. Phosphate-buffered saline (PBS) pH 7.4, high-glucose Dulbecco’s modified Eagle’s medium (DMEM) without Phenol red, Advanced DMEM, Glutamax, Penicillin/Streptomycin (10,000 U/mL), and non-essential amino acids (10X) were obtained from Gibco, Life Technologies. Fetal bovine serum (FBS), trypsin, Calcein AM (CAM), and propidium iodide (PI) were supplied by Sigma-Aldrich. Hoechst 33342 was purchased from ThermoFisher Scientific. Paraformaldehyde was supplied by VWR. Phalloidin-Tetramethylrhodamine B isothiocyanate (TRITC) was purchased from Merck and Mowiol 4-88 reagent was supplied by CalBiochem. All purchased materials were used without further purification.

### GelMA Synthesis

Gelatin methacrylamide was synthesized according to Shirahama *et al.*(49) Briefly, 10% w/v type A gelatin (300 Bloom) solution in carbonate-bicarbonate (CB) buffer (0.25 M, pH 9) was prepared. The flask was plunged into an oil bath at 50 °C without stirring for 20 min. Afterward, the mixture was stirred vigorously for 1 h until complete dissolution. Subsequently, MAA was added dropwise to the gelatin solution (gelatin/MAA feed ratio was 1:1.1). MAA excess was calculated with respect to free amino groups of gelatin, as reported by Van Den Bulcke *et al.*(15) The reaction was continued under stirring at 50 °C for 3 h and then the pH was readjusted to 7.4 to stop the methacrylation reaction. Final solution was dialyzed against distilled water using dialysis tubing at 37 °C inside an incubator with slight orbital shaking. Dialyzate was changed four times within 24 h to remove salts, methacrylic acid, and anhydride. The purified product was frozen at −80 °C overnight, lyophilized for 1 week, and finally stored at −20 °C protected from light until further use.

### Nuclear Magnetic Resonance Spectroscopy

The successful derivatization of gelatin was confirmed by nuclear magnetic resonance (NMR) spectroscopy.^50^^1^H NMR spectra in a solution of pure gelatin and GelMA were acquired at ambient temperature on a Bruker AV-400 spectrometer operating at a proton Larmor frequency of 400.16 MHz. To prepare the samples, unmodified gelatin and GelMA solutions were prepared at 25 mg/mL in deuterium oxide with TMSP as an internal standard (1 mg/mL). Data were processed using MestReNova software.

All high-resolution magic angle spinning (HRMAS) NMR spectra were acquired at room temperature (RT) on a Bruker Avance NEO 400 spectrometer operating at a proton Larmor frequency of 400.13 MHz and equipped with a 4 mm double-resonance (^1^H, ^13^C) gradient HRMAS probe. Samples were swollen in deuterium oxide and chemical shifts were referenced to TMSP (as an internal reference). The gels were mechanically stable at the moderate magic angle spinning rate of 4 kHz used in all the HRMAS experiments and no sample instabilities resulting from centrifugation-related phenomena were detected.

### Degree of Functionalization: TNBS Assay

The degree of functionalization (DF) was defined as the percentage of amino groups (from lysine and hydroxylysine) derivatized in GelMA.^51^ The quantification consisted in the determination of remaining free amino groups using TNBS based on Habeeb method^52^ and Lee *et al.* modifications.^53^ All samples were prepared in duplicate for the following protocol. Briefly, GelMA and gelatin samples were separately dissolved in 0.1 M CB buffer pH 8.5 (concentration for gelatin: 1 mg/mL; concentration for GelMA: 5 mg/mL), and 50 μL of each gelatin solution was pipetted into 96-well plates. Then, 25 μL of 0.1% w/v TNBS was added and the microwell plate was incubated for 2.5 h at 37 °C in the dark with gentle shaking.^50^ Next, 25 μL of 10% w/v SDS solution and 12.5 μL of 1 M HCl were added to each sample to stop the reaction. Absorbance was measured at 330 nm with a microwell plate reader Multiskan Go (Thermo Scientific). A glycine standard curve was used to calculate the amino group content with standard sample solutions prepared at 0, 0.16, 0.32, 0.48, 0.64, and 0.72 mM. All glycine solutions underwent the same TNBS procedure as GelMA samples. DF was calculated as shown in formula 1:
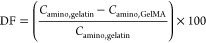
1where *C*_amino,gelatin_ and *C*_amino,GelMA_ are the amino contents (in mmol/g) in gelatin and GelMA, respectively.

### Hydrogel Preparation

GelMA hydrogels were generated by photoinduced gelation of the GelMA macromer in aqueous media—PBS or DMEM—and a photoinitiator. GelMA-SH hydrogels were fabricated in the same way but including a thiolated PEG in the solution. All gelatin solutions were prepared at 6% w/v GelMA macromer and 0.1% w/v photoinitiator as final concentrations either in PBS or in DMEM.

For hydrogels in PBS, a stock solution of 1% w/v photoinitiator was prepared by dissolving I2959 in neat methanol. Next, the required amounts of freeze-dried GelMA macromer and I2959 solution were mixed together and dissolved in PBS at 37 °C protected from light. Incubation and stirring took place for 1 h until complete dissolution of GelMA macromer was achieved. Then, for GelMA-SH hydrogels, 5% w/v 4-arm PEG thiol solution in PBS was added to the mixture at a final functional group ratio of methacrylamide/SH of 1:0.5. PDMS cylindrical molds were fabricated for swelling and atomic force microscopy (AFM) experiments (Figure S1). 130 μL of gelatin mixture was directly poured into cylindrical molds (*D* = 6 mm, thickness = 3 mm) for swelling hydrogels, while for AFM experiments, 120 μL of the mixture was poured into thinner molds (*D* = 10 mm, thickness = 1 mm) mounted on top of glass slides. Exposure to UV light (320–390 nm, 10 mW/cm^2^) was initiated after 20 min of physical gelation at RT using an OmniCure S2000 UV Lamp leading to the final photopolymerized hydrogels. The duration of the exposure to UV light is indicated in the name given to each material; that is, for GelMA-150, the mixture was exposed to UV light for 150 s.

To fabricate hydrogels in culture medium, the same protocol was followed, substituting PBS for DMEM. Phenol red-free DMEM was employed in order to avoid undesirable potential effects during photopolymerization such as UV light attenuation and thus gradients in photocrosslinking.^54^ Thus, hydrogels were prepared in high-glucose DMEM without phenol red or FBS and supplemented with 1% Penicillin/Streptomycin and 1% v/v of non-essential amino acids (“simple medium”). All processes were performed under sterile conditions.

### Hydrogel Characterization

#### Mechanical Testing: Atomic Force Spectroscopy

Young’s moduli of hydrogels were characterized by AFM nanoindentation in contact mode using a NanoWizard 3 AFM module (JPK Instruments AG, Germany) equipped with an optical inverted microscope (Nikon-Eclipse). AFM experiments were performed with qp-BioAC-CB1 probes (Nanosensors, Switzerland) with a nominal spring constant of 0.3 N/m. Calibration of the cantilever was assessed prior to mechanical testing, and the thermal noise method was used to measure the exact spring constant before each experiment. Measurements were performed in PBS, and a Petri dish heater (JPK Instruments AG, Germany) was used to keep the sample at 37 °C. As described above and to facilitate handling and provide a precise positioning of the hydrogel, this was directly cured on a glass substrate. The ensemble was incubated in PBS at 37 °C for 24 h and then placed inside the Petri dish filled with tempered PBS. Indentations were performed at a rate of 2 μm/s up to a force setpoint of 1 nN. Three to four force maps were recorded per sample with an 8 × 8 pixel resolution over a 10 × 10 μm area. The Young’s modulus was calculated using the AFM software (JPK SPM Desktop—Nanowizard) by fitting the Hertz model to the acquired force curves approximating the tip as a 15° cone. Three samples of each condition were tested for calculations of means and standard deviations.

AFM measurements for DMEM hydrogels were performed identical to PBS protocol, substituting this buffer for simple medium.

#### Swelling Behavior

Gel fraction was determined for GelMA hydrogels. For PBS hydrogels, samples were frozen in liquid nitrogen after crosslinking and lyophilized overnight. Freeze-dried gels were weighed (*W*_d1_), followed by swelling in PBS at 37 °C for 24 h. Liquid excess was gently removed from samples with a KimWipe paper, and subsequently, hydrogels were frozen in liquid nitrogen, lyophilized overnight, and weighed again (*W*_d2_). Gel fraction (%) was calculated by formula 2
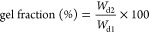
2To calculate the mass swelling ratio, hydrogels were first incubated in PBS at 37 °C for 24 h. Then, samples were blotted with a KimWipe paper to remove the excess of buffer solution and weighed (*W*_s_). Next, hydrogels were frozen in liquid nitrogen, lyophilized overnight, and weighed again (*W*_d_). Mass swelling ratio (g/g) is defined as
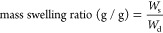
3Three replicate samples of each condition were tested for all calculations.

Swelling behavior for DMEM hydrogels was characterized using the same protocol as in PBS but substituting this buffer for simple medium. Swelling steps were performed under sterile conditions.

### Cell Culture

Caco-2 cells were cultured in flasks in Advanced DMEM supplemented with 10% v/v FBS, 1% Penicillin/Streptomycin, and 1% v/v of non-essential amino acids (“complete medium”). Cells were kept in an incubator at 37 °C and 5% CO_2_ (standard conditions) and passaged at 90% confluence. Macromer solutions containing GelMA or GelMA and multifunctional thiol crosslinker were prepared in the simple medium. PDMS molds were used to fabricate GelMA and GelMA-SH hydrogels (10 mm diameter, 1 mm thick). Upon photocrosslinking, hydrogels were transferred to 24-well plates and immersed in DMEM at 37 °C. After 24 h of swelling, cells were seeded on top of the hydrogels to generate the cell culture. Caco-2 cells were trypsinized, counted, and re-suspended in a complete medium at a density of 2 × 10^6^ cells/mL. Caco-2 cells were seeded at a density of 5 × 10^5^ cells/cm^2^ on top of the 6% w/v GelMA and 6% GelMA-1% 4PEGSH hydrogel discs. After 24 h, cell-seeded hydrogels were transferred to new 24-well plates and cultured for 14 days under standard conditions exchanging the complete medium every 2–3 days. Three hydrogels were seeded for each condition.

#### Cell Viability

Cell viability was evaluated using a Live/Dead staining protocol at days 1, 7, and 14 of cell culture. Cell-seeded hydrogels were incubated with 2 μg/mL CAM and 4 μg/mL PI in the complete medium for 25 min under standard culture conditions. After staining, hydrogels were transferred to new 24-well plates and turned upside down (cell layer downward) in order to get better fluorescence images. The complete medium was added to every well and viability was monitored using an inverted fluorescence microscope (Leica DMi8). Fluorescence images were processed using ImageJ software and manually thresholded to quantify cell viability.

#### Phalloidin Staining and Visualization of Actin

After 14 days of culture on the GelMA-based hydrogels, cells were fixed with 4% paraformaldehyde for 20 min at RT. After rinsing three times with PBS, hydrogels were incubated with Phalloidin-TRITC (2 μg/mL) and Hoechst 33342 (*ca.* 40 μg/mL) to stain F-actin and cell nuclei, respectively. Hydrogels were incubated with the staining mixture for 60 min at RT while protected from light. Then, they were washed with PBS and placed with the cell layer downward onto a glass coverslip coated with Mowiol. 25× water immersion objective was used for cell imaging.

#### SEM Imaging of Cell-Seeded Hydrogels

For SEM imaging of cell-seeded hydrogels cultured for 14 days, the aforementioned paraformaldehyde fixation protocol was followed. Then, hydrogels were dehydrated through a graded ethanol series (30–50–70–90–96–96–100–100–100%) for 10 min each and dried at RT overnight. Finally, hydrogels were mounted on stubs using conductive carbon tape, sputter-coated with 14 nm of palladium, and imaged with a scanning electron microscope (CSEM-FEG Inspect 50, FEI) using 10 kV of acceleration voltage and spot size 3.

### Data Analysis and Statistics

All data are expressed as mean ± standard deviation. The software used in graph plotting and statistical analysis was OriginPro 2020 software (OriginLab). Shapiro–Wilk normality test and equality of variances between data sets were studied before significance testing. Student’s *t*-tests and one-way ANOVAs with Tukey’s post hoc tests were used to determine significant differences (*p* < 0.05). Non-parametric Mann–Whitney analysis was performed with non-normally distributed data.

## Results and Discussion

### Functionalization of Gelatin

As mentioned in the Introduction section, the incorporation of methacryloyl groups into gelatin is a frequently used method that enables the polymer to be crosslinked upon irradiation with UV light. In this study, experimental parameters and optimized conditions to functionalize gelatin were adjusted as described in Shirahama’s method.^49^ The successful methacrylation of primary amines present in lysine and hydroxylysine residues was confirmed by ^1^H NMR spectroscopy^50^ (Figure S2). The presence of the methacrylamide groups was evidenced by the signals at 5.4 and 5.7 ppm assigned to the vinyl protons and the peak at 1.9 ppm belonging to the methyl groups. Furthermore, the characteristic lysine signal at 3.0 ppm almost disappeared in the spectra of GelMA, pointing out the reaction of the ε-amino groups with the MAA. TNBS assay results showed that free amines were transformed to methacrylamide groups with an 84% yield. It is worth mentioning that the reaction does not involve arginine residues. Therefore, the RGD motifs remain intact and GelMA retains good cell adhesive properties.^16^

### Preparation and Characterization of Cell-free Hydrogels in PBS: Modulation of UV Irradiation Time

**Figure 1:**
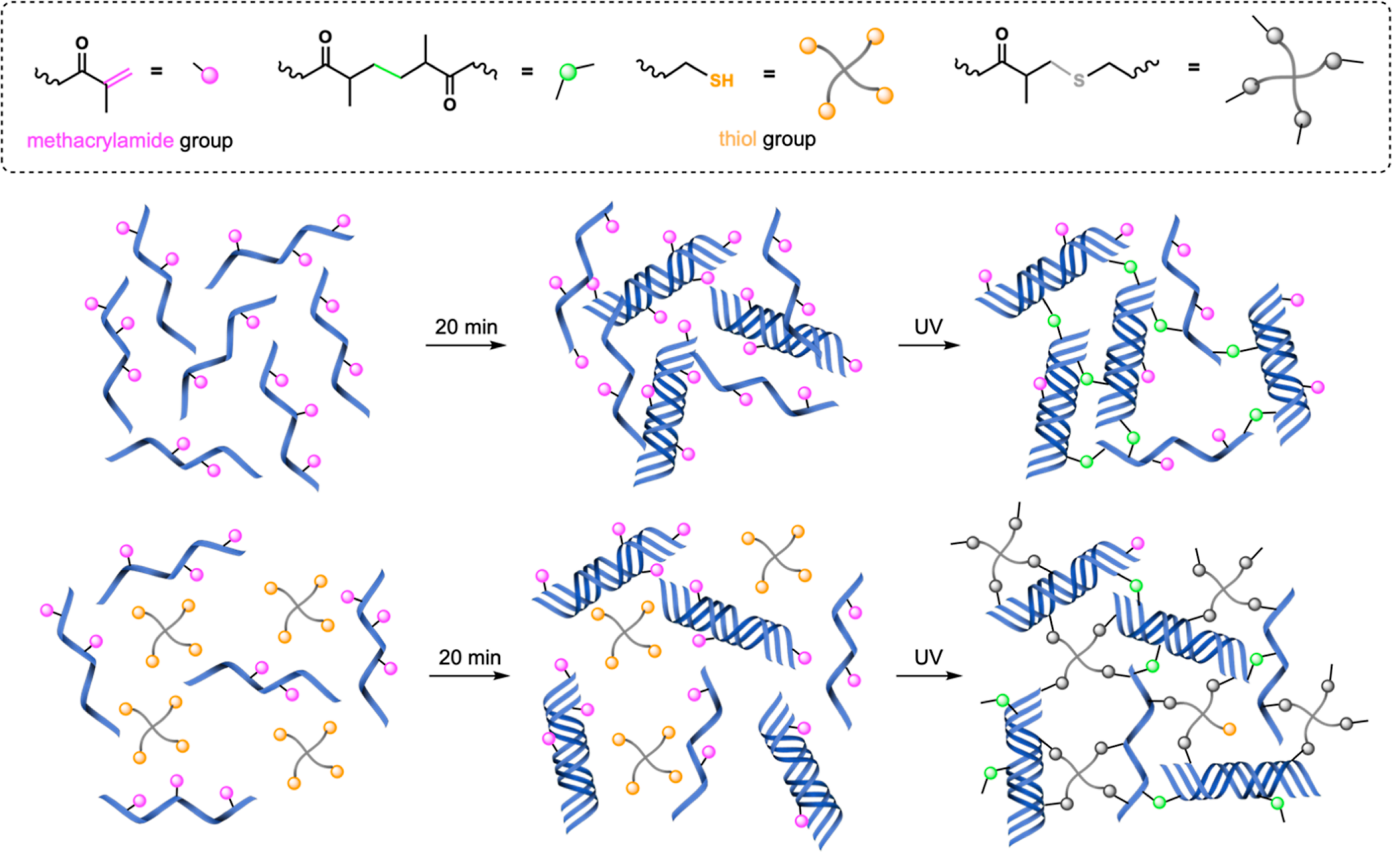
Crosslinking procedures for GelMA and GelMA-SH hydrogels. Physical gelation at RT takes place during the 20 min incubation period.

In the present work, we chose the photoinitiator system I2959 since it has demonstrated good cell viability over a reasonably long culture period.^55^ As for the GelMA concentration, it was fixed at 6% w/v to ensure biocompatibility and cellular response.^56^ Thus, for all gelatin derivative solutions, warm mixtures dissolved in PBS were poured into PDMS molds and kept at RT protected from light for 20 min before UV irradiation forming a physical gel. During this time, random coil GelMA chains are held together by hydrogen bonds, resulting in triple helix formation and hence in physical gelation (Figure 1).^57^ In this work, photopolymerization is performed after physical gel formation process as it is known to lead to stiffer covalently crosslinked networks with superior mechanical properties.^14^

Herein, GelMA and GelMA-SH hydrogels were prepared by exposing the photoinitiator-containing precursor formulations to actinic light. On one hand, light-induced GelMA network formation proceeds following a chain-growth mechanism. This procedure has been extensively studied owing to the on-demand photopolymerization possibilities.^58,59^ The other photocrosslinking strategy consisted in the introduction of thiol-methacrylamide chemistry, for which GelMA-SH hydrogels were developed, including a synthetic thiolated PEG as a crosslinker. In this case, irradiation of the photoinitiator with actinic light generates radicals that can initiate the chain-growth photopolymerization and also the step-growth mechanism of the network formation (mixed-mode crosslinking). This click and step-growth nature of the thiol-ene radical reaction typically yields a higher conversion of the functional groups over chain-growth reaction.^56^ Regarding the selection of a four-arm thiol as a multifunctional crosslinker, it is known that high crosslinking agent functionality renders suitable polymerization efficiencies and good ultimate network architectures.^60^ Additionally, the functional group ratio between the double bonds and sulfhydryl nucleophiles is not trivial either. Excessive thiol or even an equimolar ratio promotes the presence of dangling structures instead of crosslinking.^61^ Consequently, GelMA-SH hydrogels were prepared at a methacrylamide/thiol ratio of 1:0.5, which corresponds to concentrations of 6% w/v GelMA and 1% w/v 4-arm thiol in water.

#### HRMAS NMR

For the chemical characterization of the hydrogels, HRMAS NMR spectroscopy was used. For GelMA hydrogels, longer exposure times led to higher conversion in the photopolymerization as proven by the decrease of signals belonging to the vinyl and methyl protons of the methacrylamide (Figure S3). The decrease was also observed for the GelMA-SH hydrogels (Figure S4), but more interestingly, in this case, a multiplet appeared at 2.98 ppm (Figure S5). This signal is downfield shifted with respect to the crosslinker methylene protons alpha to the thiol group at 2.73 ppm. Consequently, the new signal can be ascribed to the same protons after thiol-ene reaction between GelMA and the PEG crosslinker and to the formation of the sulfide linkage. Thus, for GelMA-SH hydrogels, photoinduced crosslinking occurs in a mixed-mode fashion, that is, through the concomitant chain-growth polymerization of methacrylic groups of GelMA and the step growth thiol-ene reaction.

#### Mechanical Testing: AFM

Unlike compressive modulus which represents bulk hydrogel properties, AFM measurements determine local surface Young’s modulus,^62,63^ thereby constituting a better approach to characterize the microscopic environment that surrounds cells, especially when seeded on a surface.

**Figure 2:**
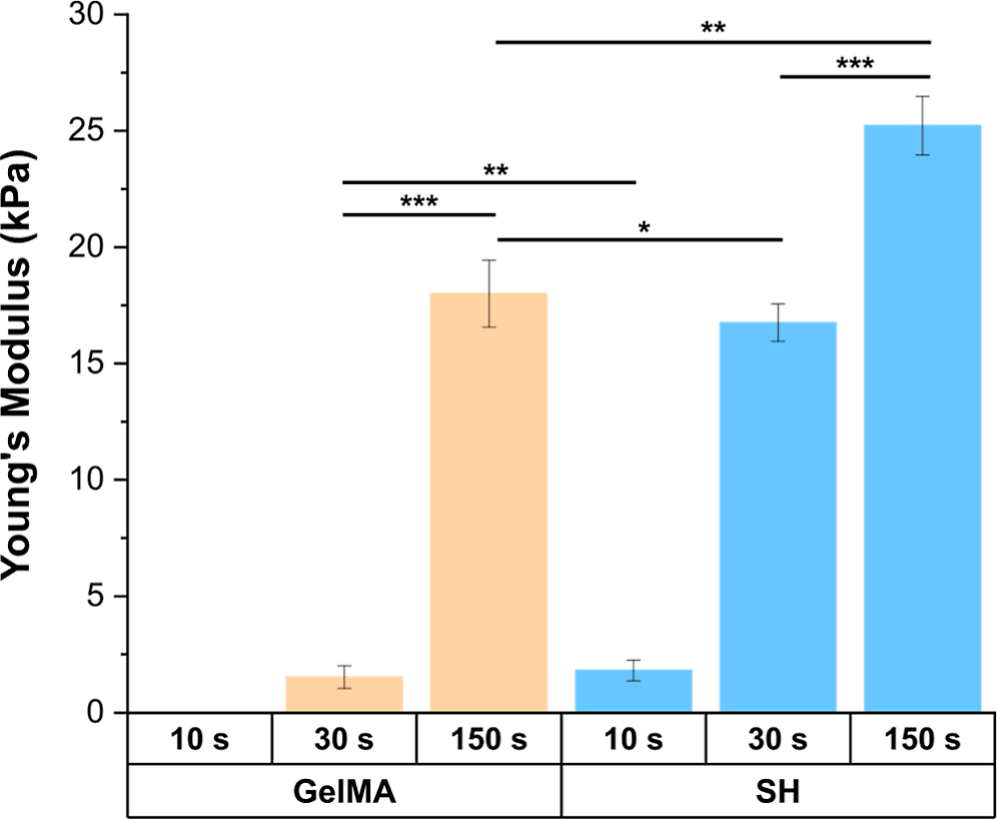
Stiffness dependence on curing time for GelMA and GelMA-SH hydrogels prepared in PBS after 10, 30, and 150 s of UV irradiation. Error bars SD. Data analysis was performed with non-parametric methods. Note: ***p < 0.001, **p < 0.01, *p < 0.05 (statistical differences *p* < 0.0001 are not drawn in the graph).

Thus, our first studies involved measuring the stiffness of GelMA-based cell-free hydrogels so as to select the best candidates for further cell culture. Since the goal of this study is to achieve good biomimetic models and cell culture is bound to the swollen state, the Young’s moduli of hydrogels were measured in PBS at 37 °C after equilibrium swelling at physiological temperature (Figure 2).

Free-radical crosslinked GelMA networks were prepared by irradiating with UV light for 10 s, 30 s, or 150 s (named GelMA-10, GelMA-30, and GelMA-150, respectively). Mechanical testing of GelMA-10 hydrogels was not possible due to the difficulties to keep its integrity during handling. GelMA-150 gels exhibited a Young’s modulus of 18 kPa, 12 times higher than that of GelMA-30, that is, 1.5 kPa. This significant increase in stiffness was also observed with the thiol/methacrylamide system (named “SH hydrogels”), but this time, shorter curing times were needed to achieve equivalent Young’s moduli to those reached in GelMA hydrogels. Remarkably, thiol click chemistry enabled a 3-fold decrease in the UV dose for moduli near 1.5–2 kPa and up to a 5-fold decrease of exposure for stiffness around 17–18 kPa, compared to GelMA system. As described above, the addition of a four-armed thiol entailed a photoinduced mixed-mode crosslinking strategy comprising the methacrylamide chain-growth polymerization and the thiol-ene reaction. Regarding the composition differences, SH hydrogels were prepared at 1% w/v of four-armed thiol, therefore yielding a final formulation with 7% w/v of polymer content. Despite this small increase in the solid content toward GelMA scaffolds (6% w/v), which might contribute to higher stiffness, the sharp increase of Young’s moduli exhibited in SH hydrogels could be probably due to the mixed-mode crosslinking, favored by the higher concentration of reacting groups and the higher mobility of four-arm PEG thiol compared to GelMA macromers.

The use of a four-arm thiol as a crosslinker was found to be a powerful tool to easily tune mechanical properties, so a deeper study was performed by varying the irradiation times from 10 to 300 s (Figure S6). Young’s moduli progressively grew up to ∼17 kPa within 30 s of UV crosslinking (1.8, 4.8, and 11.7 kPa after 10, 15, and 20 s of UV irradiation, respectively), and crosslinking, therefore stiffness, tended to saturate for longer UV times (25.2 and 28 kPa for SH-150 and SH-300, respectively). We hypothesize that either there is no longer a significant amount of unreacted methacrylamide groups (all of them have already reacted) or steric hindrance makes difficult to crosslink more gelatin chains, even though there are still photoreactive methacrylamide groups available.

In biological samples, elastic modulus is the associated measure of matrix stiffness for pathological stages in colorectal tumors. Thus, studies revealed that the normal tissue stiffness is around 1 kPa,^64^ while colorectal cancer tissue displays a stiffness variability according to the degree of disease.^26^ Nebuloni *et al.* used nanoindentation measurements by atomic force microscopy to measure the Young’s moduli of CRC samples derived from three donors to obtain median values of ca ∼55, ∼14, and ∼23 kPa.^65^ In a comprehensive study with measurements on CRC tumor samples obtained from 106 donors, Kawano and co-workers reported that the elastic modulus of colorectal cancer tissue is strongly related with the tumor size and its metastatic features.^26^ As reported by Kawano *et al.*, T4 stage in TNM classification (18 donors) was described with a median of 13.8 kPa, an interquartile range (IQR) from ∼8 to ∼30 kPa, and a range from 5.58 to 68.0 kPa. Since the Young’s moduli of all gelatin-based hydrogels prepared in the present work match with Kawano’s and Nebuloni’s results, a more detailed mechanical analysis was performed (gel fraction and swelling behavior results are discussed in the following sections) with these four scaffolds: GelMA-30, GelMA-150, SH-10, and SH-30. The stiffness of these scaffolds corresponds either to that of the healthy colorectal tissue—GelMA-30 (1.5 kPa) and SH-10 (1.8 kPa)—or to the stiffness of a CRC tissue—GelMA-150 (18.0 kPa) and SH-30 (17 kPa)—while allowing the comparison between materials of similar stiffness but different crosslinking strategies.

In accordance with these AFM measurements, the thiol-methacrylamide system enabled to shorten times of exposure to UV light, hence setting up an attractive basis for future 3D culture: the potential cell damage could also be drastically minimized. This fact is supported by a recent work of Isik *et al.*,^66^ who revealed severe differences in cell viability between hydrogels irradiated with UV light (23 mW cm^–2^) for 1 and 2 min.

To sum up, finely tuned hydrogels have been successfully prepared and a precise control over stiffness has been achieved with high reproducibility. Furthermore, UV exposure has been revealed as an easy-adjustable and influential parameter to create a wide platform of bioscaffolds with elastic moduli that covers the range from healthy (∼1 kPa)^64^ to tumoral tissues.

### Gel Fraction and Swelling Properties

**Figure 3:**
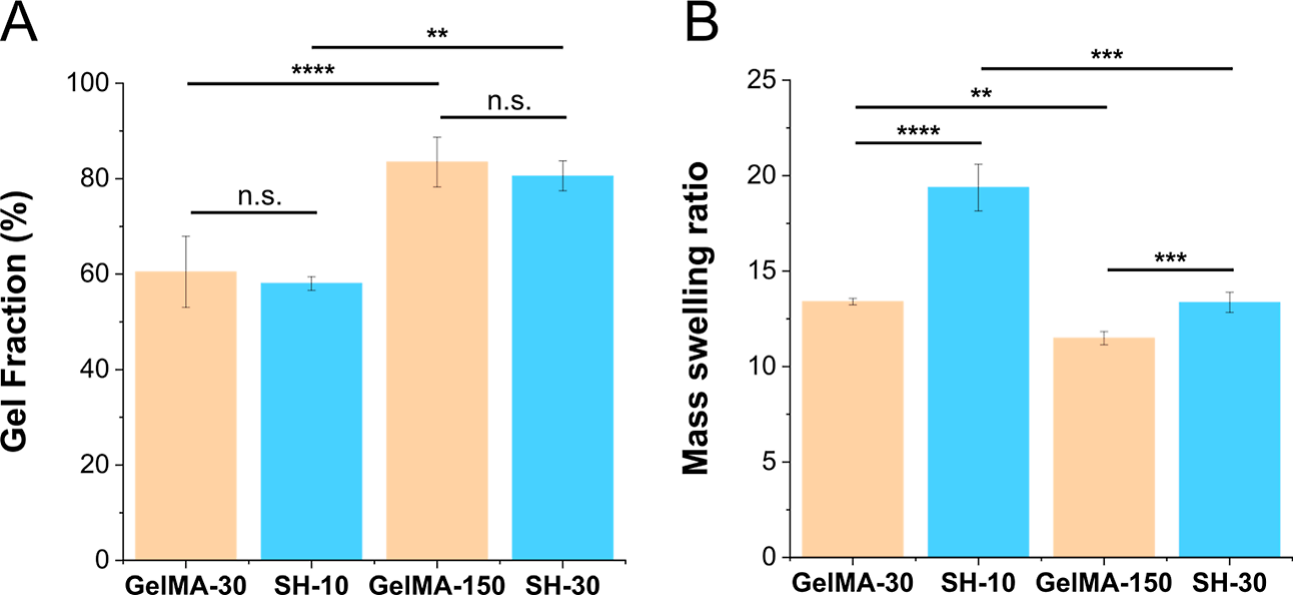
Swelling characterization in PBS: gel fraction (A) and mass swelling ratio data (B). Error bars SD. Note: ****p < 0.0001, ***p < 0.001, **p < 0.01, *p < 0.05.

As shown in Figure 3A, there was no significant difference between the gel fractions of GelMA-30 and SH-10 hydrogels with values of 60.5 and 58.1%, respectively. These values indicate that part of the crosslinkable molecules of the formulation were not bounded to the network and leached out. Increasing the dose of light during curing led to higher gel fractions with values of 83.5 and 80.6% for GelMA-150 and SH-30, respectively, demonstrating a higher incorporation of molecules into the hydrogel network. Comparable gel fractions to our results have been reported in the literature for related materials.^24,34^ Briefly, we found that hydrogels prepared using different strategies, GelMA and GelMA-SH, but leading to similar stiffnesses also presented similar values of gel fraction.

Regarding the swelling behavior, in contrast, SH-10 exhibits a ∼1.5-fold higher mass swelling ratio than GelMA-30 (Figure 3B), despite having similar gel fraction and stiffness values. The mass swelling ratio is smaller in hydrogels cured with higher doses of light, indicating a higher degree of crosslinking. When comparing the two hydrogels with a higher curing dose, the mass swelling ratio of SH-30 is also ∼1.2-fold higher than that of GelMA-150. The presence of hydrophilic pegylated crosslinkers in the GelMA-SH networks can account for the increased swelling that is less marked in networks with higher degrees of crosslinking.

These results are in agreement with previous reports from Bertlein *et al.*,^34^ who recorded different swelling behaviors depending on the type of network. GelMA hydrogels produced *via* free-radical photopolymerization would form heterogeneous networks^36,67^ with lower swelling capacity, while thiol-ene systems are associated with a more homogeneous distribution of crosslinking density and higher swelling properties.

### Preparation and Characterization of Cell-Free Hydrogels in Standard Culture Conditions

#### Mechanical Testing: AFM

The same as in PBS characterization, Young’s moduli of hydrogels were measured at 37 °C while immersed in DMEM after 24 h swelling at physiological temperature.

Notably, stiffness results for DMEM hydrogels were considerably different from those of PBS ones. Besides, trends were opposite for GelMA and SH scaffolds: the former networks exhibited lower Young’s moduli in DMEM (1.1 and 5.5 kPa for GelMA-30 and GelMA-150, respectively), while the latter increased their stiffness (3.1 and 22.1 kPa for SH-10 and SH-30, respectively) with respect their homologous hydrogels generated under same irradiation conditions in PBS (see Figure 2 above). Recently, Monfared *et al.*(54) have described the effect of cell culture media on radical photoreactions. Their study of the free-radical photohydrogelation of poly(ethylene glycol)diacrylate confirmed that gel stiffness when prepared in cell culture media was not significantly different from the control prepared in water. They also investigated a thiol-ene photoclick system using a four-arm functionalized poly(ethylene glycol)-norbornene with dithiothreitol, reporting that the control sample, processed in aqueous solution without any culture medium, had a higher storage modulus than the hydrogels prepared in cell growth media. In the light of Monfared results and ours, it could be suggested that besides the reaction mechanism and the added culture media, the type of macromer is not a trivial issue when addressing the influence of the culture medium in radical photopolymerizations.

#### Gel Fraction and Swelling Properties

**Figure 4:**
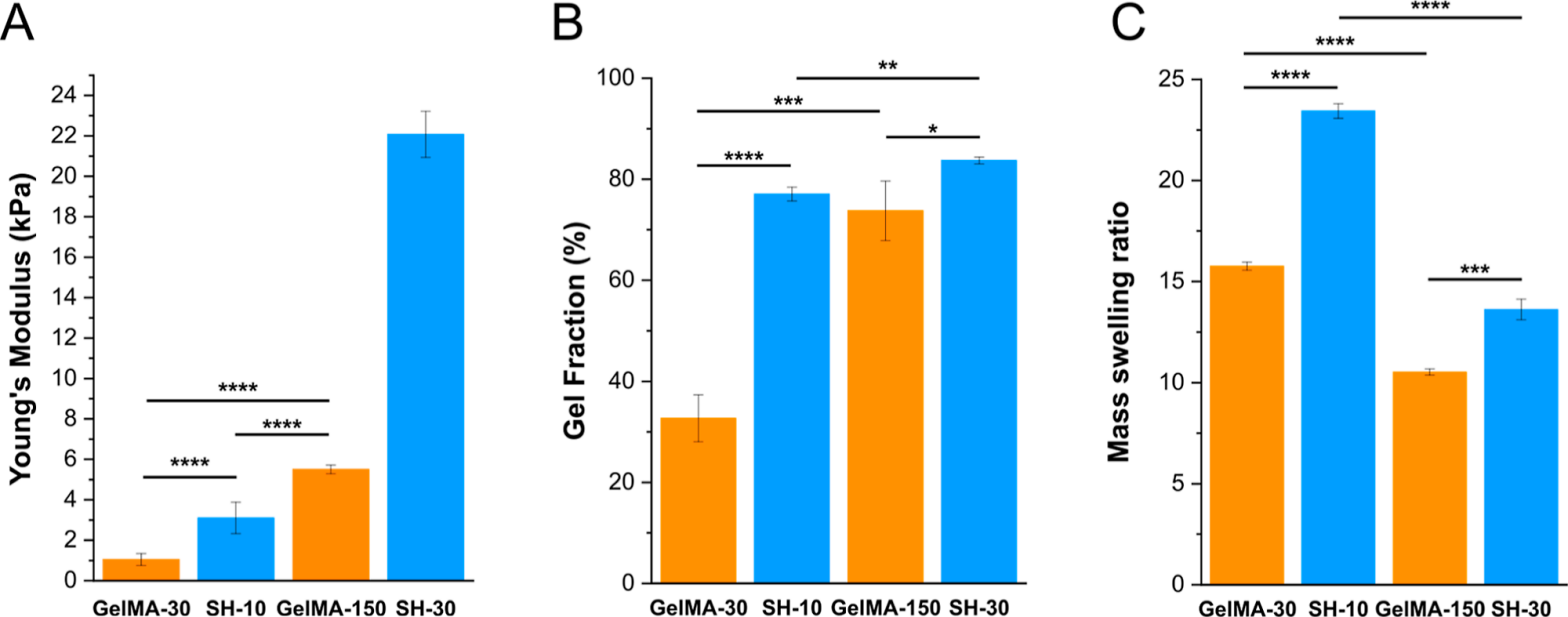
Characterization of GelMA and SH scaffolds prepared and swollen in DMEM in terms of stiffness (A), gel fraction (B), and mass swelling ratio (C). Error bars SD. Note: ****p < 0.0001, ***p < 0.001, **p < 0.01, *p < 0.05.

Regarding gel fraction and swelling properties, significant differences were found between hydrogels prepared in PBS (Figure 3A,B) and DMEM (Figure 4B,C). GelMA-30 DMEM hydrogels had a noteworthy reduction (1.8-fold) in the gel fraction compared to their PBS hydrogel analogues, indicating that there are fewer macromer units linked to the network. Consequently, and given this less crosslinked network, samples showed a higher mass swelling ratio (1.2-fold). These results are in accordance with the stiffness decrease shown in the previous mechanical testing section. For GelMA-150, similar values of gel fraction were obtained for PBS and DMEM hydrogels; however, mass swelling ratio did exhibit some differences, resulting in a 1.1-fold reduction. On the other hand, SH-10 scaffolds presented an unpredicted behavior with a gel fraction 1.3-fold higher than that of PBS hydrogels, while the swelling ratio was also 1.2-fold higher. This large swelling capacity might be explained by a higher incorporation of hydrophilic PEG-thiol chains. Regarding SH-30 hydrogels, gel fraction and swelling behavior exhibited no differences between preparations in PBS and DMEM. Taking all these results into account, it can be confirmed that scaffolds with lower degrees of crosslinking are more strongly influenced by the preparation medium.

### Cell Culture

#### Cell Adhesion and Proliferation

**Figure 5:**
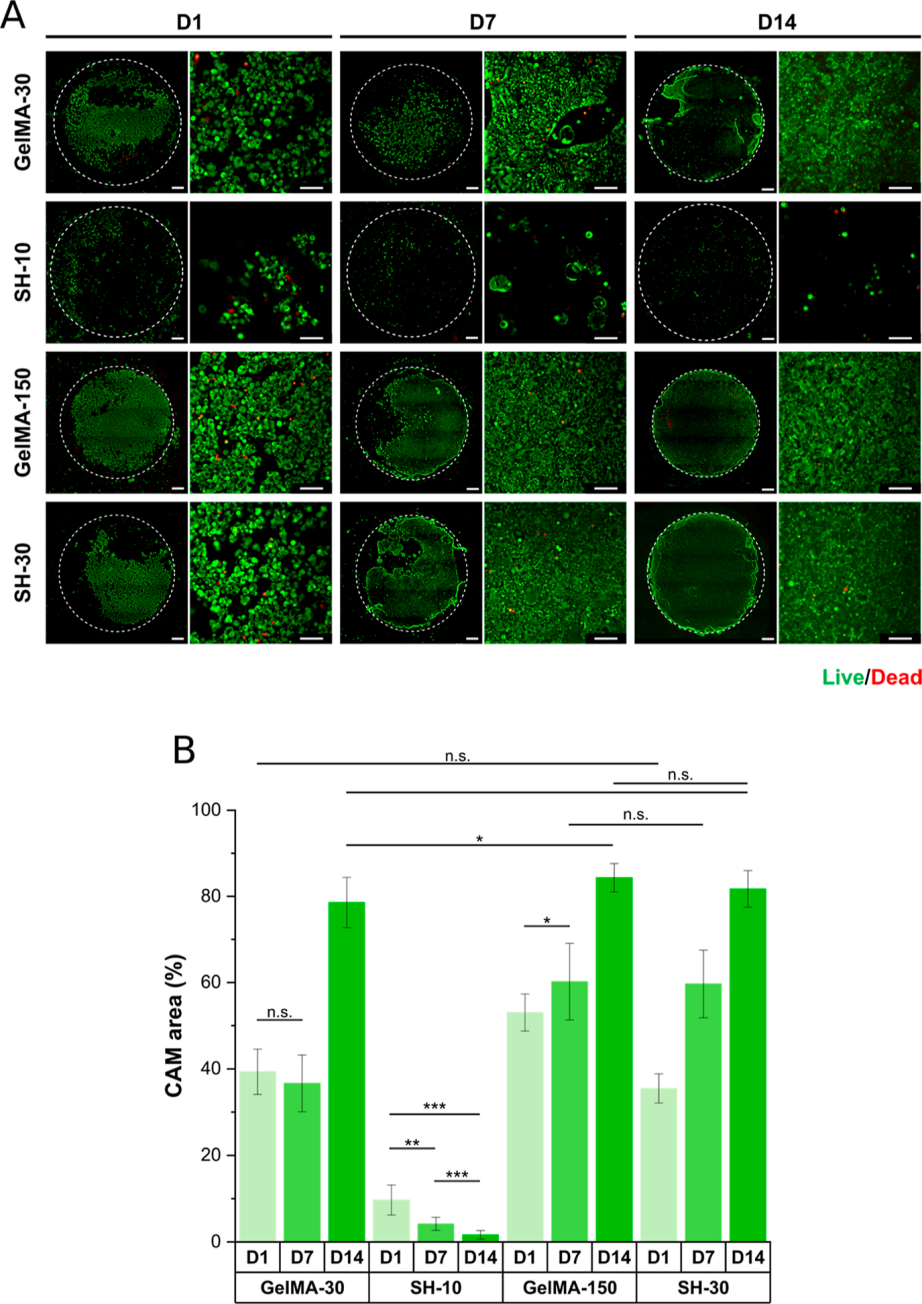
Culture of Caco-2 cells on GelMA-based hydrogels over 14 days. (A) Live/dead micrographs of Caco-2 evolution on GelMA-30, SH-10, GelMA-150, and SH-30 scaffolds on day 1, day 7, and day 14 of cell culture. Hydrogel surface outlined by a dashed white line. (B) Area percentage of alive calcein (green) stained cells from micrographs of Caco-2 on GelMA-based scaffolds on day 1, day 7, and day 14 of cell culture. The scale bar represents 1 mm and 100 μm for 5× and 20× micrographs, respectively. Error bars SD. Note: ***p < 0.001, **p < 0.01, *p < 0.05 (statistical differences *p* < 0.0001 are not drawn in the graph).

Caco-2 cell line was chosen to study the biological response toward different substrates in terms of stiffness and surface chemistry. Cell seeding was performed on top of GelMA and SH hydrogels to mimic the epithelial model. Cell viability was evaluated using a CAM/PI staining at 24 h, 7 days, and 14 days after cell seeding. As shown in Figure 5A, a cell attachment and surface coverage increase were observed on GelMA-30, GelMA-150, and SH-30 scaffolds over time, achieving a confluent monolayer over the hydrogel surface after 14 days of cell culture. These three different substrates enabled an excellent cell proliferation, but the culture evolution was different for each condition. Caco-2 cells showed the best substrate adhesion after 24 h on GelMA-150 hydrogels compared to GelMA-30 and SH-30 (*p* < 0.0001) (Figure 5B), suggesting a better surface performance for cell attachment for GelMA-150. Focusing on GelMA hydrogels, the distinct curing times applied for GelMA-30 and GelMA-150 free-radical-crosslinked networks led to differences in stiffness and swelling (Figure 4) but also in terms of cell culture behavior. Thus, GelMA-150 exhibited a higher gel fraction (74%) with a higher incorporation of GelMA macromer chains and therefore a greater number of RGD functional groups than GelMA-30 (gel fraction: 33%), pointing out that cell-binding domain density influences cell attachment. This finding is consistent with previous research showing that abundant RGD ligand presentation results in improved Caco-2 cell proliferation in 2D.^68,69^ Apart from the RGD concentration, stiffness is also a relevant parameter in cell adhesion. In fact, it is well known that stiffer substrates correlate with a greater cell adhesion.^68^ As previously described, gel fraction, RGD density, and stiffness are closely related to each other and in this case, GelMA-150 (5.5 kPa) showed a stiffer network than GelMA-30 (1.1 kPa). Taken together, the highest substrate adhesion observed in GelMA-150 hydrogels was probably due to a combination of both factors: RGD concentration and stiffness. On the other hand, SH-30 scaffolds contained a pegylated four-armed thiol within the network, yielding the highest stiffness (22.1 kPa) among all conditions studied. Focusing only on this 4-fold increase in stiffness over GelMA-150 (5.5 kPa), a better cell adhesion for SH-30 could be expected; however, cell attachment after 24 h was significantly reduced (*p* < 0.0001). These results demonstrate that substrate stiffness affects not only cell attachment but also surface chemistry and hydrogel composition.

Despite the initial differences in cell adhesion after 24 h, all conditions led to the generation of a monolayer at day 14. GelMA-30 maintained cell surface coverage after 7 days (37%), strongly increasing and almost achieving an 80% surface covered after 14 days. GelMA-150 increased significantly cell surface coverage after 7 (*p* < 0.05) and 14 (*p* < 0.0001) days, reaching a covered area of 84%. SH-30 hydrogels showed a prominent cell proliferation and surface coverage after 7 days (60%), equating that of GelMA-150 and ending up with non-statistical differences at day 14.

In contrast, SH-10 hydrogels exhibited a poor cell adhesion after 24 h (10%), decreasing until 2% of cell surface covered at day 14 and showing small, rounded, and isolated cells. This phenomenon could be correlated to the swelling behavior. It is relevant to recall that the “mixed-mode” mechanism leads to a more homogeneous crosslinked network with higher swelling capacity. According to swelling results explained in the previous section, SH-10 scaffolds exhibited the highest mass swelling ratio (Figure 4C). The SH-10 hydrogels also showed a significantly higher surface area in cell culture which may mean that the concentration of RGD domains per unit area is lower and as a result, few biological binding motifs were available for Caco-2 cells to attach. Additionally, and given the non-adhesive nature of PEG chains for cell culture, with no intrinsic biological activity, we hypothesized about these polymeric chains present in the four-armed thiol also having a detrimental effect on cell adhesion. Since SH-10 hydrogels were photoirradiated for only 10 s compared to 150 s for SH-150, it is presumable that in SH-10, more non-adhesive PEG chains were not crosslinked through their all four anchor points (thiol groups). Consequently, more non-crosslinking segments—non-adhesive dangling PEG chains—might have mobility to get access to the surface where the interaction with cells takes place.

#### Immunofluorescence Staining and SEM Imaging

**Figure 6:**
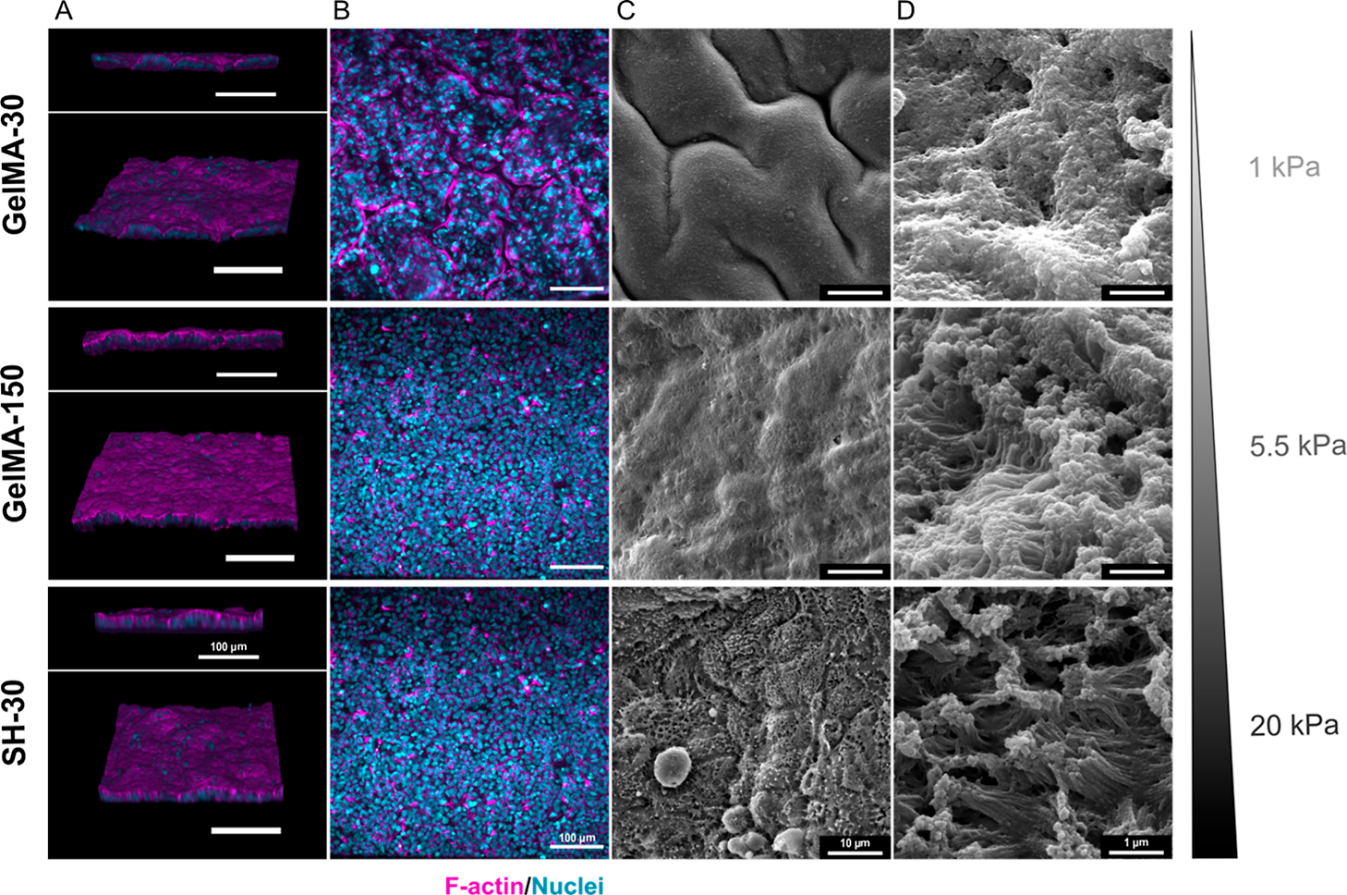
F-actin and nuclei were stained with Phalloidin-TRITC and Hoechst, respectively. 3D projection images (A) and confocal cross-sectional Z-stack (B) of Caco-2 confluent monolayer on GelMA-30, GelMA-150, and SH-30 hydrogels. SEM images of microvilli structures at day 14 at 5000× (C) and 50,000× (D) magnification.

As described above, Kawano *et al.* reported that the stiffness for normal colorectal tissue is around 1 kPa, while they found a median (IQR) CRC tissue stiffness of 13.8 (∼8–∼30) kPa. In our work, GelMA-based hydrogels prepared in DMEM enable to simulate physiological tissue stiffness (∼1 kPa for GelMA-30) and cancer tissue stiffness (∼5 and ∼22 kPa for GelMA-150 and SH-30, respectively). Representative fluorescence and SEM micrographs of the intestinal epithelium grown on GelMA-based hydrogels for 14 days (Figure 6) showed the presence of continuous polarized undulating villus-like intestinal structures, with an apical F-actin (magenta) containing brush border for GelMA-30 cultures. Likewise, the formation of basal crypts (Figure 6C) and tightly packed apical microvilli (Figure 6D) are also suggested by SEM images. Nevertheless, even though GelMA-150 and SH-30 presented F-actin expression in the apical cell area (fluorescence images Figure 6A), they did not express organized-actin macrostructures or undulating villi-like protrusions, displaying light domes for both GelMA-150 and SH-30. Furthermore, changes in apical microvilli structure were observed with increasing stiffness when analyzed at higher magnification SEM images, suggesting a loss of compactness as rigidity increases. The differential organization of actin structures depending on substrate stiffness observed in this work is consistent with the literature since ECM stiffness is critical for normal tissue development and homeostasis. Cell adhesion and signaling are strongly modulated by rigidity of the ECM to such an extent that a stiff ECM can influence tissue polarity and hence compromise tissue organization.^70^ According to these results, we can conclude that GelMA-based hydrogels are a suitable material to generate a physiological and pathological gut model.

## Conclusions

Given the outstanding potential of gelatin-based hydrogels to form custom-tailored biomimetic materials, there are plenty of functionalization approaches and modifiable parameters such as crosslinking mechanism, photoinitiator and macromer concentration, and UV dose, among others. The present study reports the fabrication and characterization of free-radical-photopolymerized GelMA hydrogels and thiol-ene photoclick systems.

Fine tuning of mechanical properties was achieved through different irradiation times for both crosslinking strategies, and in particular, a noticeable reduction of curing time was reported with the introduction of thiol chemistry. SH hydrogels exhibited higher swelling capacity than GelMA scaffolds prepared in either PBS or DMEM; however, the absolute values of gel fraction, mass swelling ratio, and also stiffness were markedly different for PBS and DMEM biomaterials. This finding sheds some light on the problems in translating the results from buffers to culture media, and our on-going research is focused on a thorough mechanical testing about it.

Regarding biological behavior, Caco-2 cells showed the best substrate adhesion after 24 h on GelMA-150, whereas they hardly adhered to SH-10 scaffolds. A good cell proliferation was achieved in GelMA-30, GelMA-150, and SH-30 since the three conditions managed to create a confluent monolayer at day 14. Finally, SEM imaging of cell-seeded hydrogels showed a proper apical-basal polarization of cells according to a villus-forming monolayer, exposing structural differences among the different substrates.

The platform of bioscaffolds generated in this work covers a wide range of mechanical properties corresponding to those from healthy tissue to cancerous stages and demonstrates the adequate differentiation of intestinal epithelium model cell line. Thus, we have stablished a basis for future studies focused on more complex stroma models and deeper epithelium characterization.

## Supporting Information Available

Hydrogel preparation protocol; ^1^H NMR spectra of the non-modified gelatin, GelMA macromer, GelMA and SH hydrogels, and free four-arm poly(ethylene glycol) thiol; and Young’s moduli of SH hydrogels (PDF)

## Abbreviations

CRC: colorectal cancer
GelMA: gelatin methacrylamide
ECM: extracellular matrix
RGD: arginine-glycine-aspartic acid
PEG: poly(ethylene glycol)
MAA: methacrylic anhydride
MWCO: molecular weight cutoff
TMSP: 3-(trimethylsilyl)propionic-2,2,3,3-*d*_4_ acid sodium salt
TNBS: 2,4,6-trinitrobenzenesulfonic acid
SDS: sodium *n*-dodecyl sulfate
I2959: 2-hydroxy-4′-(2-hydroxyethoxy)-2-methylpropiophenone
4PEGSH: 4-arm poly(ethylene glycol) thiol
PDMS: poly(dimethylsiloxane)
PBS: phosphate-buffered saline
DMEM: Dulbecco’s modified Eagle’s medium
FBS: fetal bovine serum
CAM: calcein AM
PI: propidium iodide
TRITC: phalloidin-tetramethylrhodamine B isothiocyanate
CB: carbonate-bicarbonate
NMR: nuclear magnetic resonance
HRMAS: high-resolution magic angle spinning
RT: room temperature
DF: degree of functionalization
SEM: scanning electron microscopy
AFM: atomic force microscopy
