# Supplementary material for: Tuning of Mechanical Properties in Photopolymerizable Gelatin-Based Hydrogels for In Vitro Cell Culture Systems
Source: ACS Appl Polym Mater. 2023 Jan 27;5(2):1487–98. doi: 10.1021/acsapm.2c01980 (PMC9926877; doi:10.1021/acsapm.2c01980)
Supplement: Supplementary file 1 — ap2c01980_si_001.pdf [file ap2c01980_si_001.pdf]

Supporting information

Tuning of mechanical properties in photopolymerizable gelatin-based hydrogels for *in vitro* cell culture systems

Regina Pamplona [†], *Sandra González-Lana* [‡,§,] *Pilar Romero* [†], *Ignacio Ochoa* [§,‖,⊥],

Rafael Martín-Rapún [*,†,‖,#], *and Carlos Sánchez-Somolinos* [*,‖,¶]

[†] Aragón Institute of Nanoscience and Materials (INMA), CSIC-University of Zaragoza, Department of Organic Chemistry, C/ Pedro Cerbuna 12, 50009 Zaragoza, Spain

[‡] BEONCHIP S.L., CEMINEM, Campus Río Ebro. C/ Mariano Esquillor Gómez s/n, 50018 Zaragoza, Spain

[§] Tissue Microenvironment (TME) Lab. Aragón Institute of Engineering Research (I3A), University of Zaragoza, C/ Mariano Esquillor s/n, 500018 Zaragoza, Spain

[‖] Centro de Investigación Biomédica en Red de Bioingeniería, Biomateriales y Nanomedicina, Instituto de Salud Carlos III, 50018 Zaragoza, Spain

[⊥] Institute for Health Research Aragón (IIS Aragón), Paseo de Isabel La Católica 1-3, 50009 Zaragoza, Spain

[#] Departamento de Química Orgánica, Facultad de Ciencias, Universidad de Zaragoza, C/ Pedro Cerbuna 12, 50009 Zaragoza, Spain

[¶] Aragón Institute of Nanoscience and Materials (INMA), CSIC-University of Zaragoza, Department of Condensed Matter Physics (Faculty of Science), C/ Pedro Cerbuna 12, 50009 Zaragoza, Spain

*Corresponding authors: rmartin@unizar.es and carlos.s@csic.es

Hydrogels preparation

For swelling experiments, 130 μL of gelatin mixture were poured into PDMS cylindrical moulds (D = 6 mm, thickness = 3 mm). The PDMS moulds were placed first, on top of a cyclic olefin polymer (COP) film which facilitated hydrogel detaching (Figure S1-B). Likewise, a new COP film was placed over the mould after the mixture was poured into the holes. The bottom glass slide was necessary to maintain a solid and resistant surface during the manipulation process while the top glass slide was used to apply some weight assuring a completely hydrogel flat surface (without meniscus). For AFM experiments, 120 μL of gelatin mixture were poured into PDMS moulds (D = 10 mm, thickness = 1 mm) mounted on top of glass slides, previously cleaned in an ozone chamber for 60 minutes and treated with a silanization reactant for 45 minutes to enhance hydrogel attaching. The top COP film and the top glass slide were placed same as previously described.

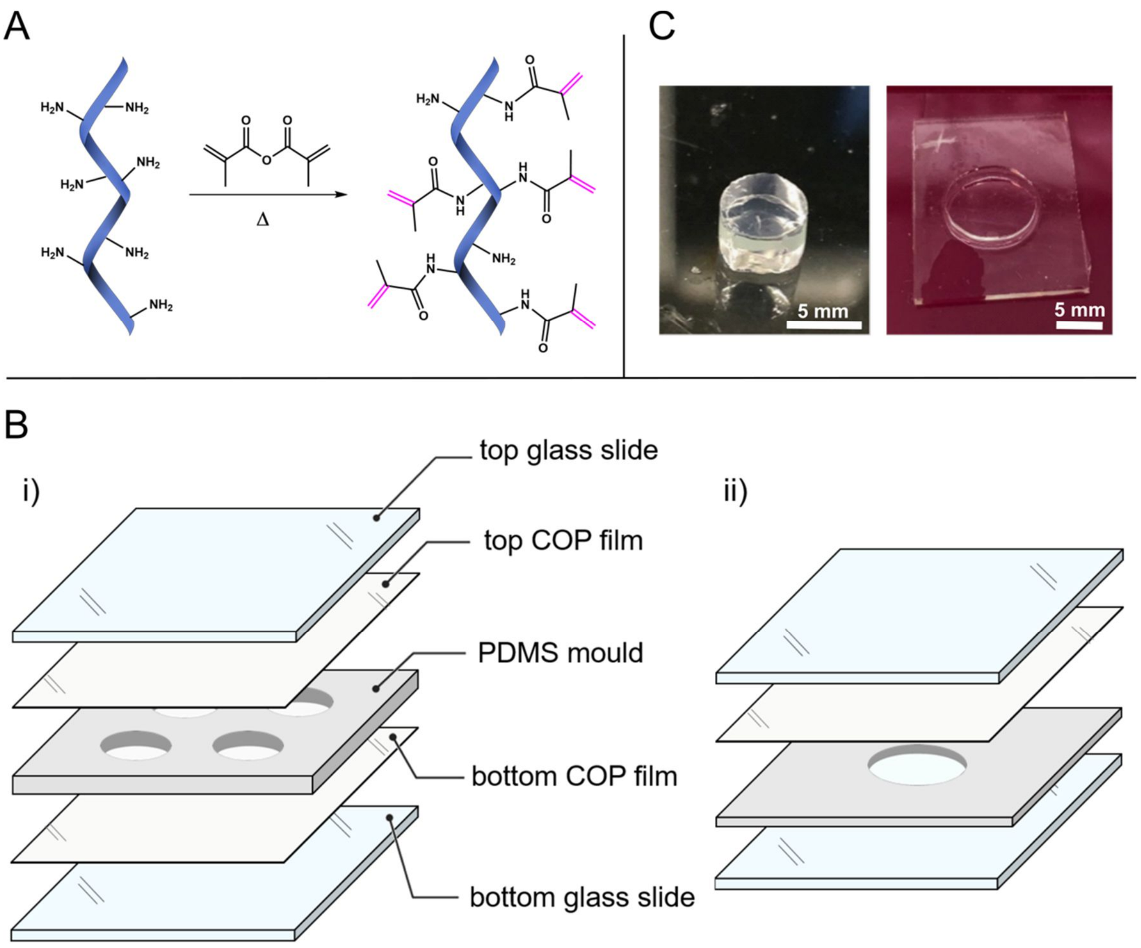

Figure S1. A) Derivatization of gelatin using methacrylic anhydride (MAA). B) Hydrogel preparation procedure: i) cylindrical hydrogels (6 mm x 3 mm) for swelling experiments were prepared according to a "sandwich" construct composed of two glass slides, two transparent COP films and the PDMS mould. ii) Disc-shaped hydrogels (10 mm x 1 mm) for AFM testing were cured directly over a glass slide (without the bottom COP film). C) Cylindrical (left) and disc-shaped (right) hydrogels.

NMR

Free radical crosslinked GelMA networks prepared by irradiating with UV light for 30 s and 150 s and photo-induced thiol-ene GelMA hydrogels irradiated for 10 s and 30 s are named as GelMA-30, GelMA-150, SH-10 and SH-30, respectively.

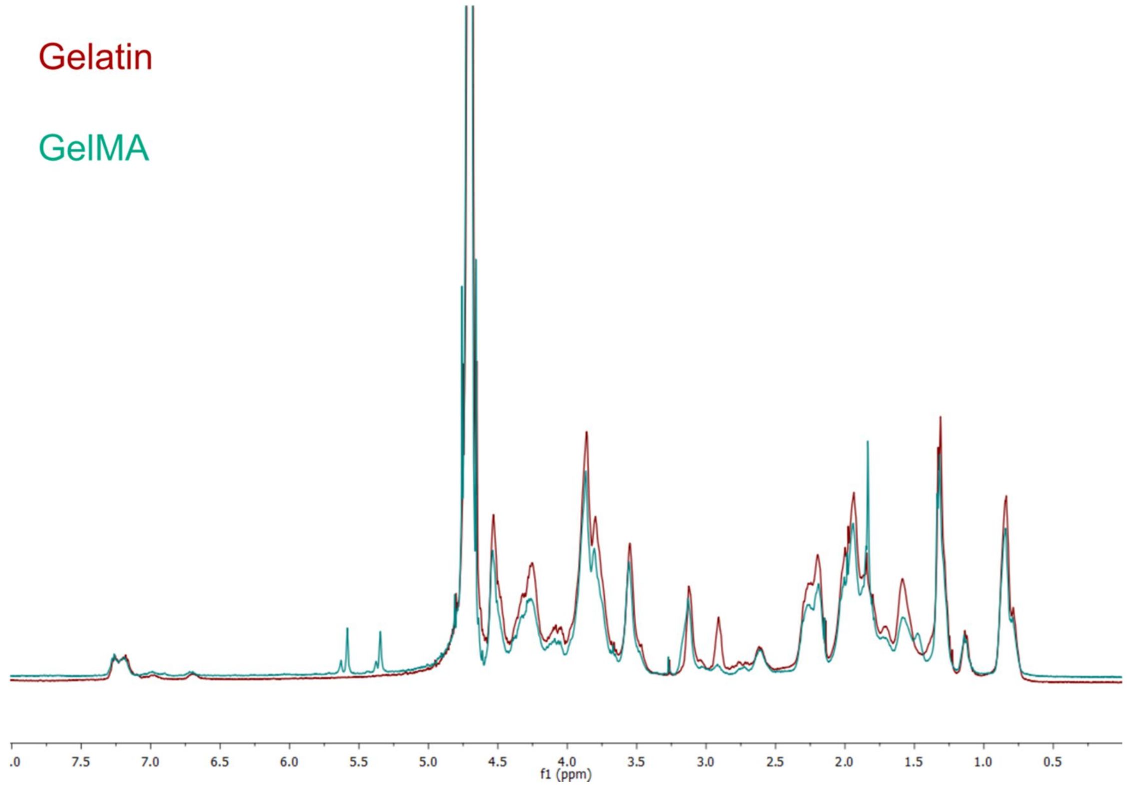

Figure S2. ^{1}H-NMR spectra of commercial gelatin (brown line) and synthesized GelMA (green line). Methacrylamide proton bands appearing at 5.58 and 5.35 ppm and the drastic decrease of the band at 2.91 ppm (ε-CH_2 unmodified lysine) are associated with the functionalization of gelatin.

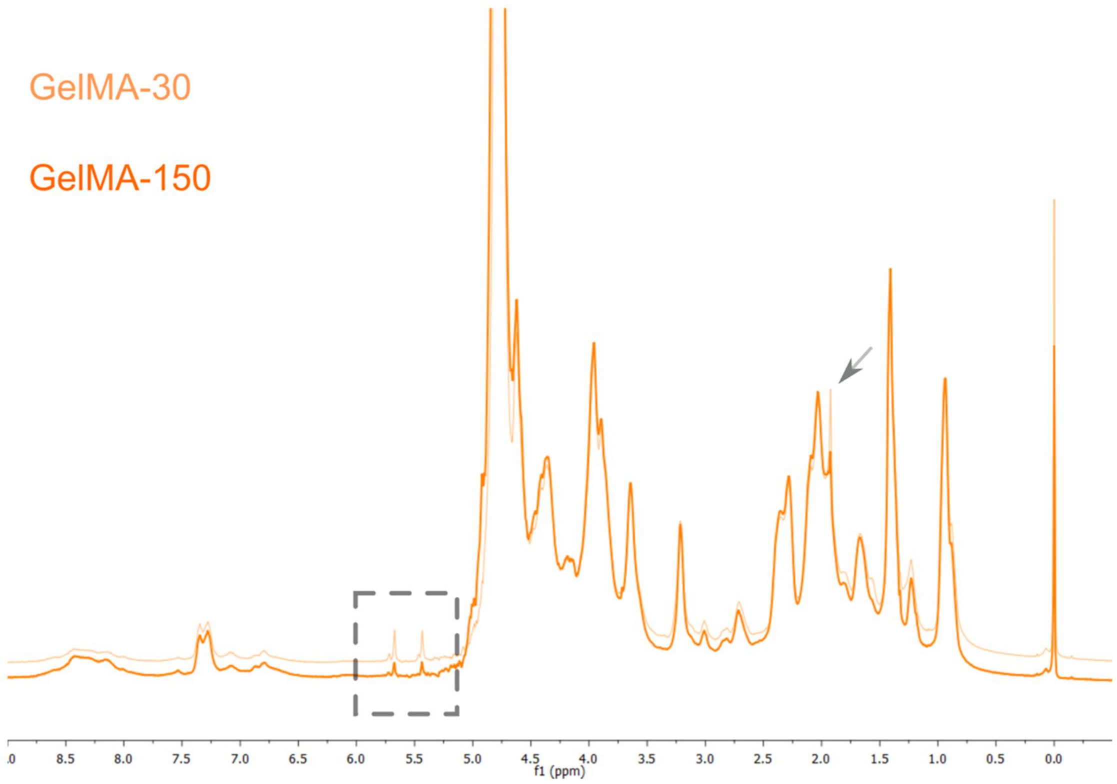

Figure S3. ^{1}H HRMAS NMR spectra of GelMA-30 (soft orange line) and GelMA-150 (dark orange line) hydrogels. Higher conversion in the photopolymerization for GelMA-150 was proven by the decrease of signals belonging to methacrylamide protons (grey dashed line).

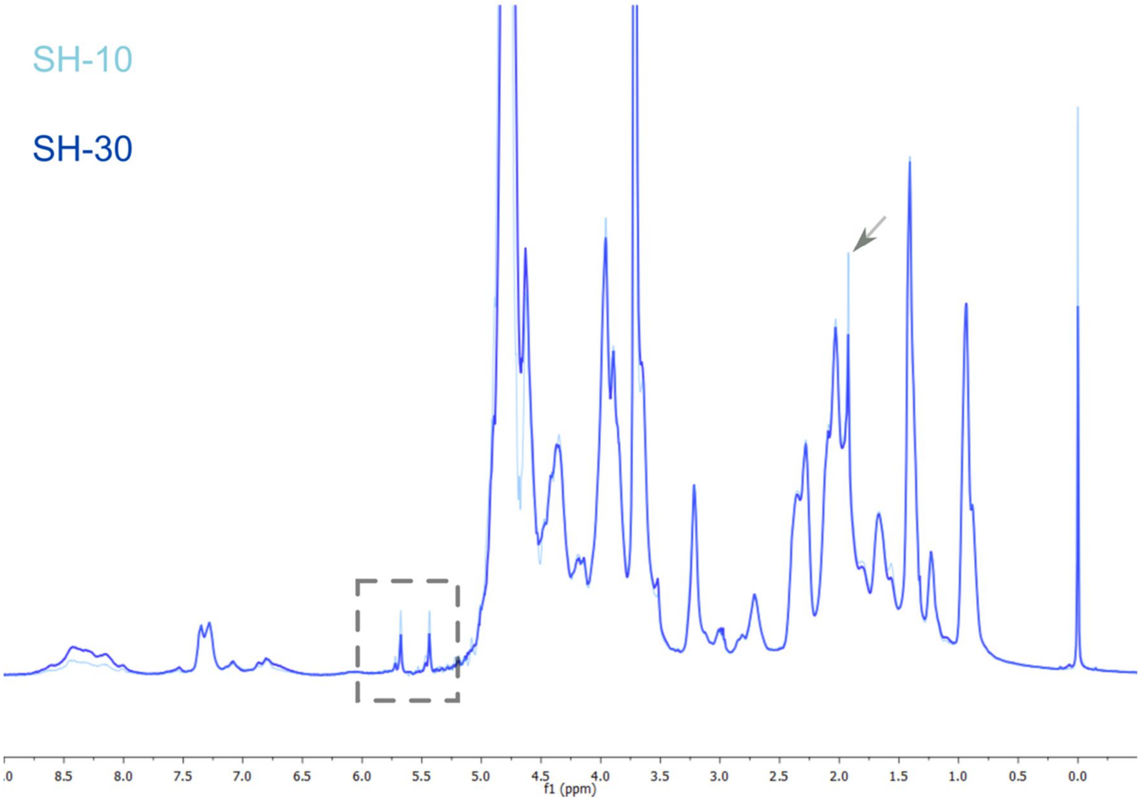

Figure S4. ^{1}H HRMAS NMR spectra of SH-10 (soft blue line) and SH-30 (dark blue line) hydrogels. Higher conversion in the photopolymerization for SH-30 was proven by the decrease of signals belonging to methacrylamide protons (grey dashed line).

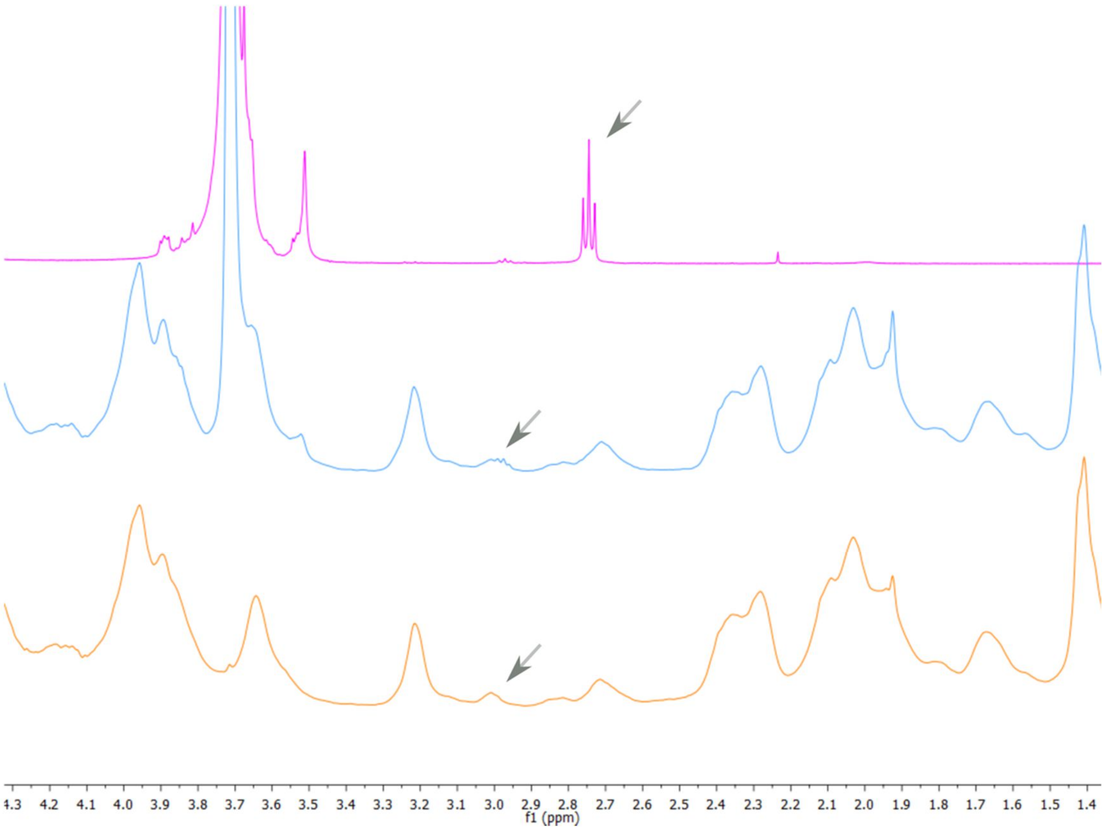

Figure S5. ^{1}H NMR spectrum in solution of free 4-arm poly(ethylene glycol) thiol (pink line) and ^{1}H HRMAS NMR spectra of SH-30 (blue line) and GelMA-150 hydrogels (orange line). The signal at 2.98 ppm is downfield shifted with respect to the crosslinker methylene protons alpha to the thiol group at 2.73 ppm.

AFM

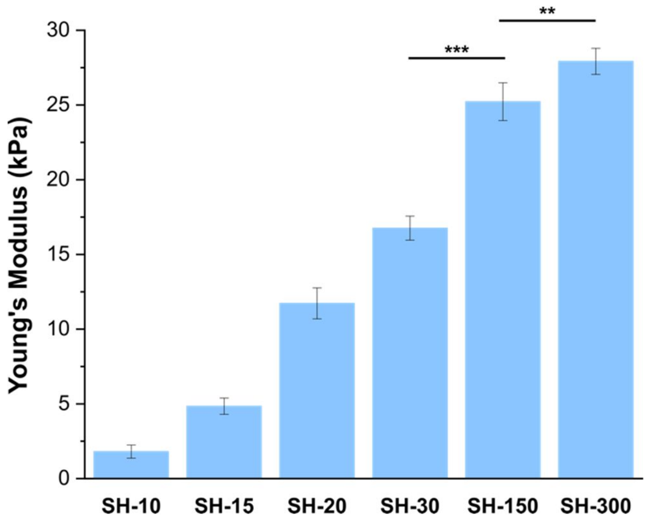

Figure S6. Young's Moduli of SH hydrogels prepared in PBS after 10, 15, 20, 30, 150 and 300 s of UV irradiation. Error bars SD. Data analysis was performed with non-parametric methods. Note: *** $p < 0.001$, ** $p < 0.01$ (statistical differences $p < 0.0001$ are not drawn in the graph).
